# Alkali-Activated Permeable Concretes with Agro-Industrial Wastes for a Sustainable Built Environment

**DOI:** 10.3390/ma18010087

**Published:** 2024-12-28

**Authors:** Shriram Marathe, Martyna Nieświec, Barbara Gronostajska

**Affiliations:** 1 Department of Materials Engineering and Construction Processes, Faculty of Civil Engineering, Wrocław University of Science and Technology, Politechnika Wrocławska 27, 50-370 Wrocław, Poland; martyna.nieswiec@pwr.edu.pl; 2 Department of Civil Engineering, NMAM Institute of Technology (NMAMIT), Nitte (Deemed to be University), Karkala 574110, Karnataka, India; 3 Department of Architectural and Construction Design, Faculty of Architecture, Wrocław University of Science and Technology, Politechnika Wrocławska 27, 50-370 Wrocław, Poland; barbara.gronostajska@pwr.edu.pl

**Keywords:** pervious concrete, alkali activation, agro-industrial waste, strength, permeability, micro-structure, sustainability, urban planning, architecture of transport infrastructure

## Abstract

This research presents a proposal for alkali-activated permeable concrete composites with the use of industrial by-products, including ground granulated blast-furnace slag (GGBS) and waste-foundry sand, as well as agro-desecrate product, i.e., sugarcane bagasse ash (SBA). GGBS and SBA served as binders, with crushed granite as coarse aggregate and waste-foundry sand as fine aggregate. The novelty of this proposal is in examining the influence of SBA, in combination with slag, on the fresh- and hardened-state properties of the proposed permeable concretes. Experiments were conducted to optimize the SBA percentage based on hydraulic conductivity and compressive and tensile strength after 28 days of air curing. The hardened density, compaction factor (workability), and saturated water absorption were also measured for all the mixes. Furthermore, the control and optimal mixes were subjected to evaluate the microstructure analysis (EDX, XRD, and FESEM) after 28 days of air curing. The mix containing 100% GGBS and 0% SBA served as the reference, with the optimal 10% SBA mix (with 90% GGBS) used for comparative analysis to understand its effect on the properties of permeable composites. The results showed positive or acceptable mechanical performance at a mix ratio of 10% SBA to 90% GGBS as binders. This study aims to enhance the understanding of the engineering behavior of alkali-activated permeable composites, facilitating the rational design of permeable pavement systems through the effective use of agro-industrial waste products, thereby conserving ecosystems while meeting engineering requirements.

## 1. Introduction

Urban development has led to significant improvements in transport infrastructure, which, while fostering economic growth, have exacerbated environmental problems such as urban runoff from surfaces (hydrological issues) and the impact of the urban heat island (UHI) effect (thermal concerns). Traditional concrete pavements significantly exacerbate these issues by hindering natural water permeation and increasing temperature at the surface [1]. Flooding during rainy seasons and periods is a common issue, compounded by the growing use of impermeable surfaces such as roads, sidewalks, plazas, and recreational areas. This problem is especially acute in metropolitan regions [2], where streets frequently feature water that remains stagnant due to inadequate drainage systems.

The resultant stagnant water disturbs daily living, presents health hazards, and may damage infrastructure. Furthermore, the UHI effect in arid seasons, marked by elevated temperatures in urban regions relative to rural areas, escalates energy consumption, exacerbates atmospheric pollution, and adversely affects human health. These difficulties underscore the necessity for efficient permeable pavement technologies to address surface runoff management. Macro-porous permeable concrete (PC) has garnered considerable interest in the construction and engineering sector as a viable solution to these issues [3]. The increasing level of research on PCs establishes a strong basis for further exploration and advancements in these materials [4]. This progress is essential for establishing sustainable infrastructure in urban settings, requiring a transition from energy-intensive construction techniques to more sustainable approaches [5]. The adoption of PC pavements can facilitate the attainment of sustainability objectives. This study focuses on assessing the engineering performance of air-cured, alkali-activated (AA) porous concrete composites derived from agro-industrial residues. This research seeks to enhance the development of eco-friendly and efficient permeable pavement surfaces by leveraging the sustainable advantages of AA materials and the potential benefits of utilizing agro-industrial by-products [6].

Generally, geopolymers and alkali-activated materials have considerable environmental advantages over Ordinary Portland Cement (OPC)-based counterparts [7,8], such as reduced carbon emissions and the capacity to include industrial waste products. By combining the porous nature of PC with the environmental friendliness of AA materials, these composites can offer effective and environmentally friendly options for urban transit infrastructure [4]. Improving sustainability by optimizing waste use in the construction industry presents a significant challenge. Although substantial research has been conducted on PCs and AA materials separately, the integration of both technologies remains largely underexplored. The potential of utilizing a combination of agro-industrial wastes in alkali-activated PC pavements has not been thoroughly investigated. This study seeks to address this research gap by evaluating the concrete engineering functionality of air-cured, alkali-activated permeable concrete (AAPC) composites [9]. This study examines incorporating readily obtainable agro-industrial by-products into porous pavement composites, employing materials such as ground granulated blast-furnace slag (GGBS), waste-foundry sand (WFS), and sugarcane bagasse ash (SBA) without utilizing traditional OPC or specialized curing techniques. This method facilitates waste management and enhances the sustainability of these composites, hence supporting the attainment of global Sustainable Development Goals (SDGs) [10].

**Figure 1:**
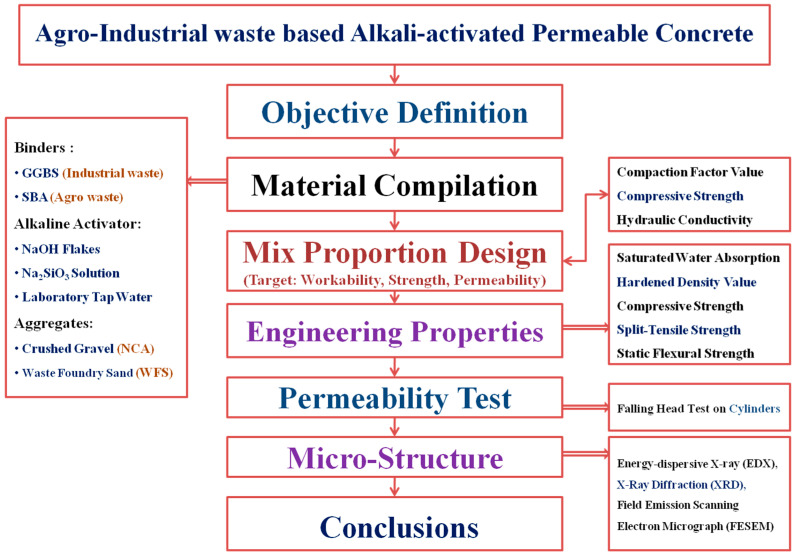
Scope of the study on agro-industrial by-product-based, alkali-activated permeable composites.

A multitude of studies have demonstrated the successful use of agro-industrial waste products in producing superior-quality alkali-activated concretes. Sheshadri et al. [11] found that incorporating WFS in pavement-quality AA mixes can improve sustainability for both the environment and the economy, permitting the incorporation of up to 20% of WFS without degrading performance, hence tackling waste management challenges in the foundry sector. Blesson and Rao [12] examined the use of agro-industrial by-products as additional cementitious components for high-strength AA composites, emphasizing the efficacy of agricultural trash, such as coconut peel ash, olive consume ash, and rice husk ash, in conjunction with industrial by-products like ultra-fine steel slag, fly ash, and GGBS as viable binder substitutes. Their review highlighted the hydraulic and pozzolanic properties of these wastes, making them feasible choices for Supplemental Cementing Materials (SCM). Further, Kathirvel et al. [13] demonstrated that substituting 20% of GGBS with SBA markedly improved compressive strength at 10% substitution rates in a specific type of geopolymer composite. This enhancement is ascribed to the augmented pozzolanic susceptibility and accelerated strength development afforded by SBA, along with enhanced durability. Similarly, many other findings also emphasize SBA’s potential as a significant element, particularly concerning slag. Consequently, various studies have demonstrated the effective use of agro-industrial wastes in producing high-quality AA composite materials; thus, there is a significant demand for research that concentrates on the engineering characteristics of PCs that integrate these by-products into environmentally friendly AA binders. This study sought to address this gap by carefully examining and recording the performance of these materials. The main objective was to formulate and enhance PC composites via alkali activation utilizing GGBS, WFS, and SBA as major ingredients. The study specifically aimed to (1) optimize the SBA proportion for satisfying the strength and permeability requirements in slag-based AAPC mixes, (2) assess the compressive strength, permeability, and tensile (split and flexural) strength of different AAPC mixes, and (3) analyze the microstructure of selected AAPC mixes through EDX, XRD, and FESEM techniques. To achieve these objectives, a rigorous test was designed. An overview of the scope of this study has been presented in Figure 1.

## 2. Materials and Experimental Methodology

### 2.1. Materials and Design Mix

**Figure 2:**
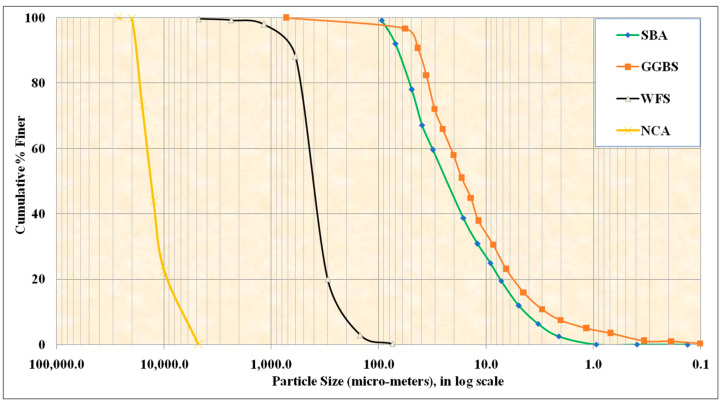
Particle size distribution.

GGBS, an industrial by-product, serves as a major binder with a chemical composition of 14.52% Al_2_O_3_, 36.89% CaO, 1.15% Fe_2_O_3_, and 38.12% SiO_2_. It has a specific gravity of 2.89 and a specific surface area of 360 m^2^/kg. The agro-waste SBA serves as a binder alternative with a chemical composition of 16.08% Al_2_O_3_, 8.10% CaO, 5.85% Fe_2_O_3_, and 59.28% SiO_2_. SBA has a specific gravity of 2.28 and a specific surface area of 462 m^2^/kg. Additionally, the physically crushed granite coarse aggregates (NCAs) with a specific gravity of 2.68 were employed for the development of AAPC test blends. Due to the reduced fine aggregate needs of PC composites, the coarse-to-fine aggregate proportion was consistently upheld at 90%:10% during the study. The industrial waste material WFS, with a specific gravity of 2.56, was employed as fine aggregate.WFS possesses distinct features and primary composition including 7.0% Al_2_O_3_, 3.0% CaO, 10.0% Fe_2_O_3_, 68.1% SiO_2_, and a specific surface area modulus of 1.91. The chosen mechanical characteristics of the coarse aggregates were as follows: NCA demonstrated an Aggregate Impact Value of 21.7%, Aggregate Crushing Value of 25.2%, Los Angeles Abrasion Value of 24.50%, and a combined Shape Index of 24.40%. The slack state density was 1508 kg/m^3^, the compact state density was 1712 kg/m^3^, and the water absorption was 0.36%. All mechanical testing of the aggregates was performed in compliance with relevant requirements. Figure 2 shows the particle size distribution (PSD) results for all the AAPC ingredients [14,15,16,17]. For the binding ingredients (GGBS and SBA), the PSD was performed using laser diffraction granulometry, and, for the aggregates (NCA and WFS), the test was performed using sieve analysis.

The alkaline activator liquid for the AAPC mixtures was composed of 98% pure NaOH solids and liquid sodium silicate (LSS) sourced from chemical suppliers. The unit mass of LSS contained 32.80% SiO_2_, 14.70% Na_2_O, and 52.50% H_2_O, with a specific gravity of 1.57, while NaOH had a specific gravity of 2.10. The solution was formulated by combining NaOH with LSS to attain a specified activator modulus (the ratio of SiO_2_ to Na_2_O) and modifying the initial water-to-binder ratio to 0.20. Thus, the ratio was modified to 0.40 during the manufacture of the mixture utilizing laboratory tap water. The alkali activator solution was permitted to mature in a sealed container for a minimum of 24 h prior to use to ensure uniform chemical composition and optimal performance of the concrete mixtures, analogous to previous studies [9,18].

The mixture design for AAPC was developed in accordance with the specifications of IRC: 44-2017 [19], aiming to achieve a target compaction factor value of roughly 0.75 and a design compressive strength of 20 MPa for conformist OPC-based permeable concrete composites. This experimental framework was modified to develop a slag-based AAPC mix, using findings from previous research [20,21]. An acceptable mix formulation was produced using 290 kg of total binder (GGBS) per m^3^ of concrete and a water-to-binder ratio of 0.40. Each mixture was formulated to sustain a minimum diffusion rate of 180 mm/min, which correlates to a Darcy’s coefficient of permeation of 3.0 mm per second. The total water content in the AA solution incorporated water from the LSS together with supplementary water to attain the requisite consistency. The activator solutions were customized for each mixture to provide a 4% Na_2_O dosage based on binder weight, ensuring a consistent activator modulus (Ms-value) of 1.25. Tap water was used in the preparation of the aqueous alkali activator solution. The total alkaline activator was calculated and consistently maintained at 143.58 kg/m^3^ over the course of the research [9,18].

Initially, GGBS functioned as the principal binder, with methodical substitutions by SBA varying from 0 to 20% in increments of 5%. The mixture containing 0% SBA functioned as the reference mixture. This methodology yielded five unique mix designs, specified as PC-0 (reference mix, comprising 100% GGBS binder), PC-5 with 5% SBA and 95% GGBS, among others. At this juncture, a compaction factor test was performed for each new mix to assess workability and verify compliance with design specifications [22]. The mix design details adopted in the current investigation is tabulated in Table 1 for clarity.

### 2.2. Experimental Methodology

Prior studies on comparable alkali-activated mixes have shown that the strength of the mixture generally increases as the curing period lengthens. However, the inclusion of SBA during the initial phases does not have a significant impact [18]. Nevertheless, as the curing period progresses, it has been seen that there is a rise in strength when there is a larger content of SBA until a certain replacement level is reached in relation to GGBS, which is considered desirable. This effect is mostly attributable to the increased creation of hydration products that correlate with particular quantities of SBA. The observed rise in strength at later ages may primarily be attributed to the high silica content, fine particle size, and the amorphous nature of SBA, as well as its pozzolanic reactivity [9,13]. Hence, this research aimed to assess the mechanical strength characteristics after a 28-day period of air curing, and the shorter durations were not considered for reporting in this article. Accordingly, for each formulation presented in Table 1, twelve cube samples (10 cm side) were prepared in the laboratory and air-cured (open-air) for 28 days, and the saturated water absorption [23], hardened density [24], and crushing strength [25] tests were carried out as per the relevant standards. The water absorption test principle involved drying the AAPC specimen in an oven until a constant mass was achieved, followed by submersion in water for 24 h to determine weight gain as a percentage of the initial dry weight. The dry density test was conducted by drying them in an oven at 100°C to 110°C until a constant mass was reached, then cooling to room temperature, weighing in air, immersing in water for 48 h, and subsequently measuring the saturated and immersed masses to calculate the density using the dry mass and the difference between the saturated and immersed masses. For the crushing strength test, the AAPC cube specimens were subjected to axial loading in a compression testing machine until failure, with the maximum load recorded to compute the strength by taking the ratio of load to the area of cross-section [18].

**Figure 3:**
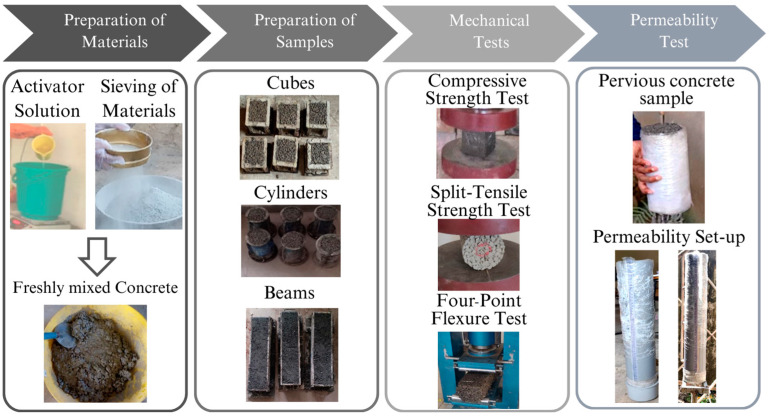
Images showing permeable concrete preparation and mechanical tests.

Additionally, 3 cylinder samples (10 cm diameter and 20 cm length) from every mix were prepared and tested for permeability [26] using the falling head permeability test method [1,27]. In this method, the specimen is set up in the test apparatus and is saturated to ensure any voids are filled completely. Later the water is passed through the top, and both the initial and final water head levels are recorded. Additionally, the time taken for a known volume of water to pass through the specimen is measured, and the hydraulic head is computed. Finally, hydraulic conductivity (permeability coefficient) is calculated using the falling head permeability equation, providing essential data for evaluating the AAPC’s permeability characteristics. Further, the four-point flexural strength test [25] on 50 cm length × 10 cm breadth × 10 cm height prism specimens and the split tensile strength test on cylinder samples [25] were also conducted after 28 days of air-curing. The flexural strength test was performed on prism specimens by placing them on two supports and applying a load at the center span until failure, allowing for the calculation of flexural strength from the recorded maximum load. The split tensile strength test involved horizontally positioning the cylindrical concrete sample in the testing machine and applying diametric compressive force until failure, with the maximum load used to calculate the splitting strength [18]. Figure 3 provides the sequence of sample preparation and major mechanical experimentation.

Afterwards, characterization examinations of the AAPC samples were performed to evaluate their microstructure, including their morphology, particulate shape, particulate confinement, chemical reaction processes, and the existence of micro-cracks. The micro-structural features of concrete have a noteworthy impact on its behavior. In order to analyze the surface characteristics and chemical composition of the products of hydration in composite mixes, Scanning Electron Microscope (SEM) imagery was combined with energy-dispersive spectrometry (EDX), as per the guidelines provided by the ASTM standard (C1723-16, 2022) [28]. In addition, the X-ray Diffraction (XRD) technique was used to identify the crystalline phases and their evolution. The micro-structural examination included examining minute samples taken from the cores of blocks of concrete that were evaluated for compressive strength after 28 days of air-curing [11,29]. The XRD analysis was conducted using a 3rd generation Empyrean instrument employing a copper (Cu) anticathode with a wavelength of 0.154056 nm. The samples obtained were ground to a particle size of less than 45 μm. These samples were then mounted on a steel stub with carbon adhesive and coated with gold for 60 s using a sputter coating device. The scanning process was performed at a step size of 0.02° 2θ/min over a total range of 5–80°, with each step taking 1 s to complete, thereby facilitating a detailed examination of the microstructure.

## 3. Results and Discussions

### 3.1. Mechanical Properties of AAPC Mixes

**Figure 4:**
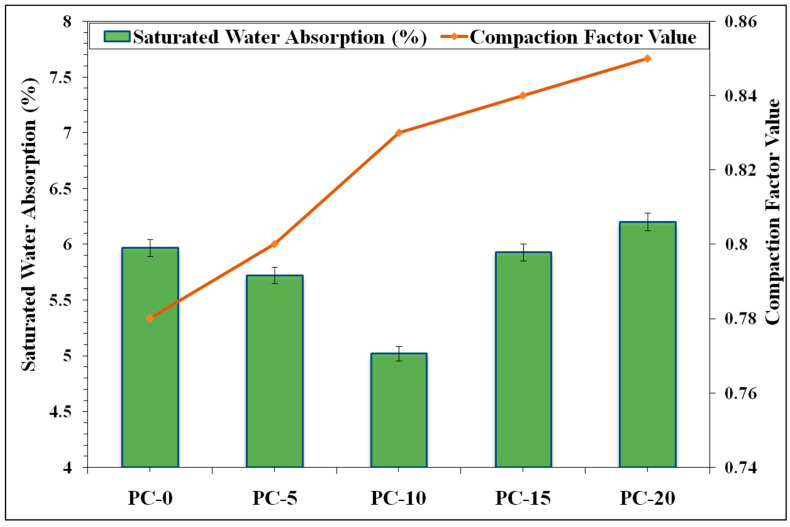
Compaction factor and saturated water absorption at varied SBA contents in AAPC.

Figure 4 presents the compaction factor values (CFVs) of the freshly prepared AAPC mixture. The established workability objective for the mixtures was a CFV exceeding 0.75, which all the mixtures successfully surpassed. The CFV rose with the incorporation of SBA as a partial substitute for GGBS. For the reference mixture PC-0, the CFV was documented at 0.78. The integration of SBA resulted in a steady rise in the CFV, attributable to the smaller particle size and greater specific surface area of SBA, which enhances packing density and diminishes internal friction within the mixture, hence improving flowability. Furthermore, prior research indicates that the use of SBA induces a diluting effect in composite mixtures, resulting in observable alterations in setting time and an associated increase in workability [30].

The outcomes of the density test reflect the compactness and the extent of void filling inside the PC matrix [31]. The baseline mixture PC-0 demonstrated a bulk density of 1897 kg/m^3^. The incorporation of SBA resulted in a marginal increase in density to 1978 kg/m^3^ at 10% replacement, suggesting that the SBA enhanced the density of the PC matrix. However, the additional rise leads to a decrement in the hardened density. The drop in density at elevated SBA contents may result from lesser specific gravity of SBA contrasted to GGBS relative to GGBS and the potential for more voids or partial hydration actions at higher substitution levels, as further evidenced by the strength results. The mean 28-day strength values for the AAPC mixtures are displayed in Table 2. All mixtures met the desired strength criterion, with the mixture containing 10% SBA (PC-10) attaining the maximum strength of 37.5 MPa. The preliminary rise in compressive strength with the SBA inclusion of up to 10% is ascribed to augmented pozzolanic activity and enhanced microstructure, resulting in a denser and more robust matrix within specific limitations. The SBA supplies additional silica [30], which interacts with the existing Ca(OH)_2_ to produce more hydration products, namely, calcium silicate hydrate (C-S-H), thus augmenting the strength [32]. Nonetheless, after 10% SBA, the strength began to diminish. This can be attributed to the dilution effect, wherein excessive substitution of GGBS with SBA diminishes the availability of the principal binding agent, resulting in incomplete hydration and reduced strength development [33].

**Figure 5:**
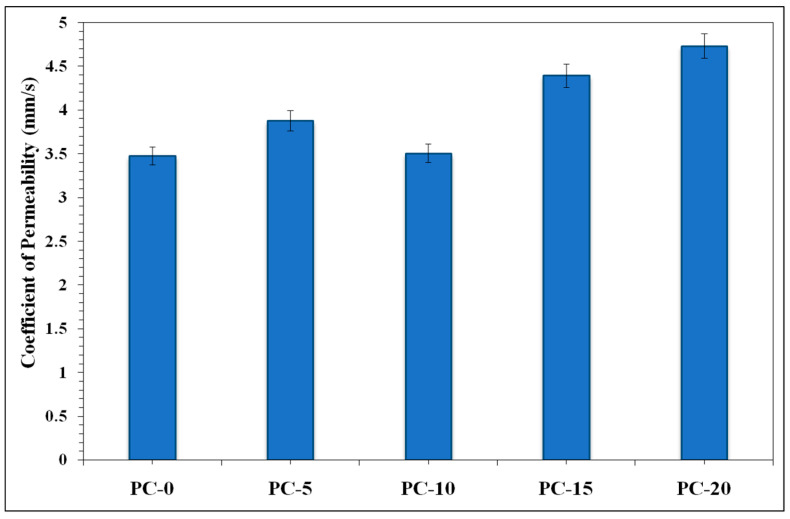
Hydraulic conductivity at varied SBA content in AAPC.

Hydraulic conductivity (permeability) was assessed for each mix and is presented in Figure 5. This value is essential for PCs as it dictates the PC composite’s ability to withstand water infiltration through its matrix, hence mitigating urban surface runoff and flooding [4]. The PC mixture that contained 10% SBA (PC-10) exhibited a marginal reduction in permeability, indicating that this substitution level preserves an ideal equilibrium between permeability and compressive strength. Further augmentation of the SBA content led to substantial enhancements in permeability. The elevated permeability values suggest that increased SBA content results in enhanced porosity and larger linked voids, hence promoting greater water flow through the concrete matrix. Nonetheless, this also correlates with a decrease in density and strength, underscoring the negotiation between permeation and mechanical qualities at elevated SBA levels [34]. Further, the tensile properties (split and flexural tensile strength) are assessed after 28 days of open air-curing, and the average results are presented in Table 2.

The optimized mix PC-10 exhibited an increase in tensile strength, with around a 5% rise in flexural strength and a 15% rise in split strength. This improvement can be attributed to the synergistic interactions of SBA and GGBS inside the AA binding system. The inclusion of SBA improves integrity by occupying voids and aiding in the formation of a denser, more cohesive substrate. The elevated silica content in SBA promotes the pozzolanic reaction, producing supplementary C-S-H gel, which improves tensile strength [30]. Further, the increased SBA dosage leads to a reduction in tensile performance; this observation is in line with the compressive strength results and the literature [18].

### 3.2. Micro-Structural Behaviour of AAPC Mixes

#### 3.2.1. Energy-Dispersive X-Ray Analysis (EDX)

This section presents the findings of the EDX analysis conducted on the AAPC under study after a 28-day period of air-curing. The presence of prominent peaks for the reference PC-0 mix at around 0.55 keV suggests a significant oxygen concentration of 34.0%. This phenomenon is prevalent because oxygen is often chemically combined with other substances, particularly silicates and oxides found in concrete. An identifiable peak at 1.74 keV with a Si content of 21.44% was observed. The silicon in concrete predominantly originates from silicates present in the slag. Ca^2+^ exhibits many peaks at various energy levels, indicating the presence of calcium as a substantial component. The most prominent peak was seen at around 3.7 keV, accounting for 15.81% of the total. The presence of calcium silicates in slag-based AAPC leads to this outcome. A significant peak was observed at around 0.3 keV, indicating the presence of carbon at a concentration of 5.36%. The spectrum has a prominent peak for aluminum, occurring at about 1.50 keV, indicating the presence of alumina at a concentration of 9.82%. Alumina is a significant constituent of slag and plays a vital part in determining the structure and strength of concrete. A minor sodium peak was seen at around 0.8 keV, accounting for about 5.39% of the total. This peak was a component of the alkaline activator, which is used to enhance the responsiveness of the binder in AAPC systems.

The results for the optimized mix (PC-10) indicate a reduction in the intensity of the EDX map, which might be attributable to the inclusion of SBA. The relative intensities of the peaks suggest that the Si peak has a lower relative intensity contrasted to the reference mix, which may be due to the higher silica content of SBA. The dominant peaks detected closely resemble those of the control AAPC mix, with 42.91% oxygen at around 0.5 keV, 14.97% silicon at 1.8 keV, 9.55% alumina at around 1.4 keV, 14.91% calcium at approximately 0.5 keV, and 3.7 keV. A significant peak is spotted at around 0.3 keV, indicating the presence of carbon (C) at a concentration of 3.32%. Iron reaches its peak value at around 0.71 keV with a relative intensity of 1.13%. Iron is often found in slag as a by-product of the steel production process using GGBS. The detection of a small peak at 1.0 keV indicated the presence of sodium (Na) at a concentration of 1.80%, most likely originating from the activator solution. The presence of C may be attributed to the organic constituents inside the SBA. The findings from the EDX study are presented in Table 3 for ease of comprehension.

After acquiring all the results, and based on the elemental composition obtained, an analysis was carried out as per the literature [35], where it was demonstrated that an increase in the Ca/Si ratio has a significantly positive effect on the mechanical characteristics of cementitious composites [36]. Similar results were presented on the pavement quality AA concrete mix with the same binder–activator composition, which also adds literature support for this finding [29]. Upon the analysis of the given attribute, it was determined that the Ca/Si ratio was 0.74 for the PC-0 mix and 0.996 for the optimum PC-10 mix. The obtained value aligns with the mechanical properties of the tested PC mixes. The optimal AAPC mixture had improved mechanical characteristics in comparison to the control mixture, evidenced by the data reported in Section 3.1. The addition of SBA resulted in a moderate increase in both compressive and tensile strength, which aligns with the Ca/Si ratio attained. Essentially, the primary distinctions among the EDX results lie in the varying intensities of the peaks. These intensities indicate alterations in elemental composition resulting from the use of various binders, such as GGBS and SBA, in each AAPC sample. The test plots exhibit minor discrepancies in the peaks of O, Si, and Ca, which may be attributable to variances in the hydration proportions of GGBS and SBA in the PC composite mixes.

#### 3.2.2. X-Ray Diffraction (XRD) Analysis

**Figure 6:**
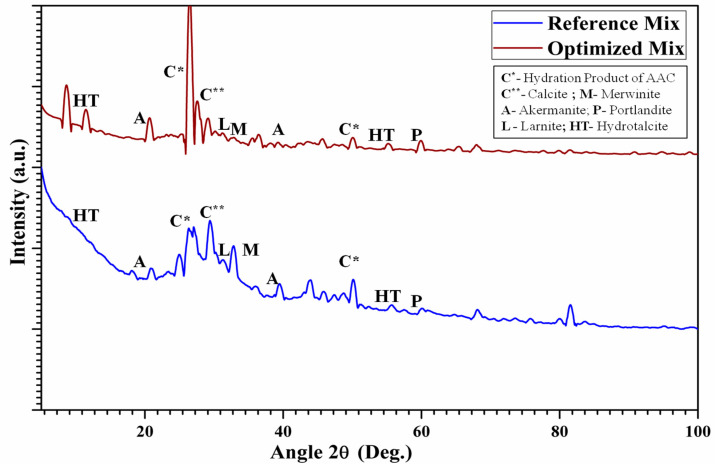
XRD patternsfor the Reference mix (PC-0), and the optimized mix (PC-10).

The XRD plot shown in Figure 6 reflects many peaks, which indicate the presence of various crystalline phases in the sample. The literature on alkali-activated composites by renowned researchers [37,38] presents distinct peaks and accompanying indications. These labels aid in the identification of the mineral phases.

The findings suggest the presence of silica-rich hydration product, as shown by many distinct peaks seen in the graph for both the control and optimized samples. The hydration phases, such as xonotlite (C-S-H) [33-0302] and chabazite (C-A-S-H) [11-0452], may be attributed to the particular reactions that occur between GGBS-SBA and an alkali activator. The dominant peak detected at an angle of around 30°, exhibiting a strong intensity, indicates the substantial presence of a hydration product. The subsequent prominent peak occurred at around 52°, having the chemical formula Ca_2_Al_4_Si_8_O_24_·12H_2_O. This phase has a superior number of peaks than xonotlite, thereby signifying its abundance in the optimized mix. Xonotlite, whose chemical formula is Ca_6_Si_6_O_18_·H_2_O, is a hydration product that can provide strength and durability when well formed. Other peaks associated with calcite (CaCO_3_) [05-0586], Merwinite [49-0643], whose chemical formula is Ca_3_Mg(SiO_4_)_2_, Åkermanite [35-0592], whose chemical formulate is Ca_2_Mg_0.9_Al_0.2_Si_1.9_O_7_, Portlandite (Ca(OH)_2_) [44-1481], Larnite (Ca_2_SiO_4_) [29-0371], and Hydrotalcite [14-0191], with an approximate chemical formula Mg_4_Al_2_(CO_3_)(OH)_12_·3H_2_O [37,39], could be perceived at their respective peaks for the comparison of the control (PC-0) and optimized (PC-10) mixes.

The XRD analysis can also detect the occurrence of secondary crystalline compounds that may contribute to augmented porosity in the PC composite matrix. In the absence of SBA, there was a notable amplification in the presence of secondary phases with inherent gel porosity, such as unreacted SiO_2_ phases. After the incorporation of SBA, the XRD showed a decrease in the intensity of peaks that corresponded to un-reacted precursor materials. This advocates that the SBA, in the presence of slag, promotes a more complete reaction and diminishes the presence of un-reacted phases that can create voids within the PC matrix. The analysis also found a decrease in the intensity of peaks corresponding to calcite in the PC-10 mixes. This reduction indicates that the SBA consumed un-hydrates during the pozzolanic reaction, leading to a denser microstructure in the optimal AAPC composite. The magnitude of an XRD peak is influenced by the quantity of a specific crystalline phase and its ability to diffract X-rays, which depends on chemical composition and absorption characteristics. Greater peak intensity signifies a higher amount of the crystalline phase, potentially indicating a greater level of crystallinity or larger crystallite dimensions. However, while peak intensity may suggest a higher degree of crystallinity, it does not directly imply a denser microstructure [13]; the crystallite size can be quantitatively assessed using Scherrer’s equation, providing further insight into the degree of crystallinity. While we did not perform such calculations in this study, the observed trends in peak intensities suggest relative variations in crystallinity among the mixes. For instance, the XRD results indicated that the reference PC-0 mix exhibited lower peak intensity compared to the PC-10 mix in the presence of SBA, suggesting a difference in the crystalline phase proportions. These findings align with previous observations [37] and highlight the influence of SBA on the microstructure [38]. More precisely, the analysis reveals the presence of many diffuse humps at about 29, 33, and 50 degrees of x-intercept. These humps are directly associated with the development of gel phases of the C-S-H type. In addition, two small peaks at about 12° and 56° 2θ are specifically associated with Hydrotalcite-group minerals, which are formed as a consequence of the reaction involving Mg^2+^ [40]. These peaks were also clearly seen in the PC-10 mix. The wide range and weak strength of these peaks indicate the gradual development of nanoscale Hydrotalcite-group crystallites. The shortening of the C-S-H humps at about 29° and 50° indicates an enhanced short-term organization of the gel [41]. Overall, the XRD pattern reveals a poorly crystalline material with distinct phases in the PC systems when combined with GGBA-SBA binders. This provides evidence for the previously reported better mechanical strength efficiency.

#### 3.2.3. Analysis of Field Emission Scanning Electron Micrographs (FESEMs)

**Figure 7:**
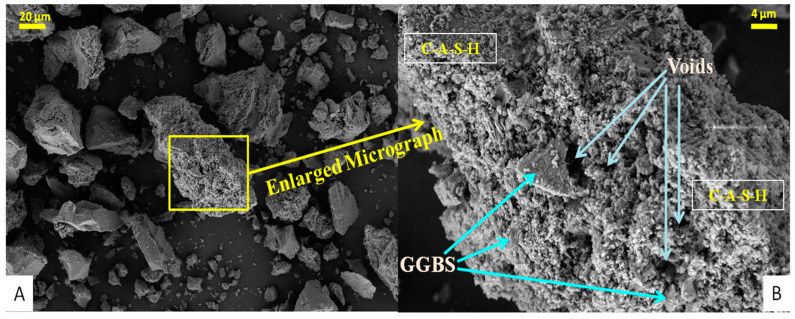
FESEM analysis plots of the control permeable alkali-activated composite (PC-0). FESEM micrograph (**A**) Normal 20 microns Scale (**B**) Enlarged 4 microns Scale.

**Figure 8:**
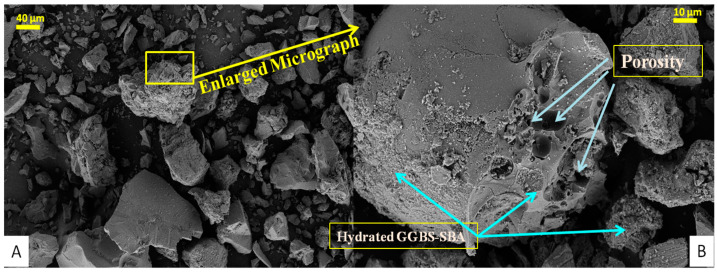
FESEM analysis plots of the optimized permeable alkali-activated composite (PC-10). FESEM micrograph (**A**) Normal 40 microns Scale (**B**) Enlarged 10 microns Scale.

The regular and magnified FESEM images of PC-0 and PC-10 samples that were taken after 28 days of air-curing are shown in Figure 7 and Figure 8, respectively. Understanding micro-scale morphological characteristics is vital for this approach [42]. Since the activator dose remains constant, the observed impact may be clearly attributed to the involvement of the agro-additive, SBA.

The un-hydrated binder particles, mostly GGBS, are shown by the white areas. The grey spaces between the particles indicate the reaction products, while the black regions represent cavities and micro-cracks [43]. Overall, based on the observed morphology, the hardened binder phase in PC-10 samples seems to be denser and more uniform in appearance. On the other hand, the sample PC-0 shows a higher number of dispersed pores, indicating a microstructure that is less dense.

The C-A-S-H phases in the microstructure, which are analogous to the C-S-H phases of concrete composites, are indicative of hydration products. These hydrated structures are intermingled with more irregularly shaped particles and clusters, which could indicate unreacted or partially reacted binding particles. The microstructure had a predominantly thick texture, interspersed with a few tiny fractures. The presence of hydrated material from GGBS (PC-0) is indicated by the rough texture and fibrous components, which contribute to the varied character of the material. On the other hand, the presence of SBA (PC-10) may make the texture smoother, as seen in the related picture. The PC-0 sample has prominent voids and imperfections on its surface, characteristic of materials with relatively low density. The surface has a coarse texture, and the arrangement of particles seems less consistent. The structure has a lower density, characterized by more prominent gaps and holes between particles in contrast to the PC-10 mixture.

The particles seemed to be clumped together, creating clusters that added to the roughness of the component. The morphology exhibited particles of diverse sizes and shapes. Certain particles had angular shapes, while others had more rounded forms. The sample’s surface had a highly textured appearance, indicating a rough and uneven surface topology. In addition, the binder environment resulting from the alkali activation of GGBS-SBA (i.e., PC-10) exhibited an amorphous and compact structure in various areas. The presence of these structures and clusters indicates that hydration products are formed by the action of the activator solution. Furthermore, the SBA not only enhanced the hydration process but also acted as filler for the pores, resulting in a denser and more cohesive material structure compared to the PC-0 combination. In Figure 8,on the right side of the micrograph, there is a rough, irregular surface with a porous appearance, which could be attributed to the presence of SBA hydrates. The surface exhibits apparent pores and spaces, which manifest as dark circular or irregular forms. Additionally, there are discernible fine fractures on the plane. The central and right sections of the FESEM display a smoother dense structure with some striations, which might be the consequence of the hydration and pozzolanic reactions occurring inside the binder milieu. The inclusion of SBA-GGBS as an additive acts as a site for nucleation, leading to the creation of more hydration products. This helps to improve the microstructure by filling in pores, reducing porosity, and enhancing particle packing. As a result, there is a more uniform distribution of particles and a more compact microstructure with smaller voids and micro-cracks. This ultimately leads to a potentially stronger composite material. The comprehensive FESEM study demonstrates an intricate interaction among the several binder components of the resulting agro-industrial AAPC composite materials [42].

### 3.3. Discussion

This study examined the evolution and efficacy of air-cured AAPC that uses SBA, an agro-industrial waste, as a partial substitute for the GGBS binder, with WFS serving as fine aggregates. The results from multiple assessments of workability, density, strength, water absorption, hydraulic conductivity, tensile characteristics, and micro-structural analyses offered insights into the effectiveness and potential of the materials in question for sustainable permeable roadway applications. The findings demonstrated that integrating SBA at optimal levels of replacement will fulfil the engineering specifications of AAPC mixtures. The ideal composition, containing 10% SBA, exhibited the most significant compaction factor (0.83), dry unit weight (1978 kg/m^3^), and compressive strength (37.5 MPa). This mixture demonstrated adequate hydraulic permeability (3.503 mm/s) and reduced absorption of water (5.02%), indicating enhanced durability. The tensile strength tests corroborated the greater efficiency of the 10% SBA mix, exhibiting improved split(2.47 MPa) and flexural (4.1 MPa)tensile strength. The micro-structural examination of the Control mix (PC-0) and the Optimized AAPC mix (PC-10) yielded insights into the documented strength advancements. The EDX analysis indicated distinct elemental peaks, with a notable increase in the Ca/Si ratio from 0.74 to 0.996, underscoring its vital contribution to strength enhancement attributed to the optimal presence of GGBS-SBA. The XRD examination revealed several peaks, indicating the existence of diverse crystalline phases and validating the presence of silica-rich hydration products in the control and optimized samples. The PC-10 mix demonstrated a reduction in peak intensity associated with calcite, indicating that SBA efficiently utilizes un-hydrated minerals during the pozzolanic process, thus enhancing the density of the microstructure in the ideal AAPC composite. The XRD results for PC-10 revealed an increase in peak intensity compared to PC-0, which may indicate a higher relative abundance of certain crystalline phases. However, peak intensity alone cannot be conclusively correlated with crystal size or mechanical performance without additional quantitative analyses, such as the application of Scherrer’s equation to calculate crystallite size. These observations are consistent with trends in the material’s mechanical performance, as referenced in the literature [13,37,38]. The FESEM morphology confirmed these observations, showing a denser and more uniform hardened hydrated binder phase (C-A-S-H) in PC-10, while PC-0 exhibited a higher quantity of dispersed voids, signifying a less dense microstructure. These observations align with the findings from additional micro-structural investigations and mechanical performance evaluations, as documented in the literature.

## 4. Conclusions

This investigation examines the performance and development of air-cured slag-based alkali-activated permeable concrete that incorporates SBA as a partial replacement for GGBS with WFS as fine aggregates. The optimized mix with 10% SBA exhibited superior properties, such as the highest compressive strength (37.5 MPa), tensile strengths (2.47 MPa split and 4.1 MPa flexural), optimal hydraulic conductivity (3.503 mm/s), and reduced water absorption (5.02%). The strength gains were supported by a denser C-A-S-H matrix with enhanced Ca/Si ratios and silica-rich hydration products as confirmed by micro-structural analyses. These results confirm that AAPC is a sustainable and enduring solution for permeable pavement settings. These findings underscore the viability of SBA as a sustainable option in alkali-activated concrete, fostering ecological and effective solutions for permeable urban construction. It can be used in urban spaces and transport infrastructure. Future research should concentrate on fiber incorporation [44], long-term durability, large-scale production challenges, and field performance to corroborate these laboratory findings, thereby facilitating the greater acceptance of sustainable construction methodologies.

## Figures and Tables

**Table 1:** Mix proportion details for 1 m^3^ AAPC preparations in kg.

Mix ID	PC-0	PC-5	PC-10	PC-15	PC-20
Waste-Foundry Fine Aggregates (WFS)	199.7	199.3	198.9	198.6	198.3
Granite Coarse Aggregates (NCA)	1881.3	1878.13	1874.89	1871.65	1868.42
Sodium Silicate Liquid (LSS)	44.207	44.207	44.207	44.207	44.207
Sodium Hydroxide Solids (NaOH)	6.583	6.583	6.583	6.583	6.583
Water	92.791	92.791	92.791	92.791	92.791
Sugarcane Bagasse Ash (SBA)	0	14.5	29	43.5	58
Ground Granulated Blast-Furnace Slag (GGBS)	290	275.5	261	246.5	232

**Table 2:** Mechanical strength properties of AAPC mixes at 28 days.

Mix ID	PC-0	PC-5	PC-10	PC-15	PC-20
% GGBS	100	95	90	85	80
% SBA	0	5	10	15	20
Compressive Strength (MPa)	32.2	34.2	37.5	30.2	27.8
Split Tensile Strength (MPa)	2.14	2.31	2.47	2.02	1.99
Flexural Strength (MPa)	3.92	4.02	4.1	3.87	3.41

**Table 3:** Compositional EDX examination of control and optimized PC composites.

Element	C	O	Fe	Na	Mg	Al	Si	S	Ca	Mn
PC-0	5.36	34.0	0	5.39	4.13	9.82	21.44	3.68	15.81	0.35
PC-10	3.32	42.91	1.13	1.8	6.5	9.55	14.97	4.87	14.91	0

## Data Availability

The original contributions presented in this study are included in the article. Further inquiries can be directed to the corresponding author.

## Abbreviations

Urban heat island (UHI); permeable concrete (PC); alkali activated (AA); Ordinary Portland Cement (OPC); waste-foundry sand (WFS); ground granulated blast-furnace slag (GGBS); alkali-activated permeable concrete (AAPC); sugarcane bagasse ash (SBA); Supplementary Cementing Material (SCM); naturally crushed granite aggregate (NCA); Sodium Silicate Liquid (LSS); Scanning Electron Microscope (SEM); energy-dispersive spectrometry (EDX); X-ray Diffraction (XRD); compaction factor value (CFV); calcium silicate hydrate (C-S-H); calcium–aluminum silicate hydrate (C-A-S-H).
